# Preparation of epigallocatechin gallate-loaded nanoparticles and characterization of their inhibitory effects on *Helicobacter pylori* growth *in vitro* and *in vivo*

**DOI:** 10.1088/1468-6996/15/4/045006

**Published:** 2014-08-05

**Authors:** Yu-Hsin Lin, Chun-Lung Feng, Chih-Ho Lai, Jui-Hsiang Lin, Hao-Yun Chen

**Affiliations:** 1 Department of Biological Science and Technology, China Medical University, Taichung, Taiwan; 2 Division of Hepatogastroenterology, Department of Internal Medicine, China Medical University Hospital, Taichung, Taiwan; 3 Department of Microbiology, School of Medicine, China Medical University, Taichung, Taiwan; 4 Bio-Medical Carbon Technology Co., Ltd, Taichung, Taiwan

**Keywords:** Helicobacter pylori, epigallocatechin-3-gallate, fucose–chitosan, gelatin, nanoparticles

## Abstract

A variety of approaches have been proposed for overcoming the unpleasant side effects associated with antibiotics treatment of *Helicobacter pylori* (*H. pylori*) infections. Research has shown that epigallocatechin-3-gallate (EGCG), a major ingredient in green tea, has antibacterial activity for antiurease activity against *H. pylori*. Oral EGCG is not good because of its digestive instability and the fact that it often cannot reach the targeted site of antibacterial activity. To localize EGCG to *H. pylori* infection site, this study developed a fucose–chitosan/gelatin nanoparticle to encapsulate EGCG at the target and make direct contact with the region of microorganisms on the gastric epithelium. Analysis of a simulated gastrointestinal medium indicated that the proposed *in vitro* nanocarrier system effectively controls the release of EGCG, which interacts directly with the intercellular space at the site of *H. pylori* infection. Meanwhile, results of *in vivo* clearance assays indicated that our prepared fucose–chitosan/gelatin/EGCG nanoparticles had a significantly greater *H. pylori* clearance effect and more effectively reduced *H. pylori*-associated gastric inflammation in the gastric-infected mouse model than the EGCG solution alone.

## 1. Introduction

Peptic ulcer disease occurs in the stomach (gastric ulcer) or in the duodenum (duodenal ulcer), which is a worldwide health problem because of its high morbidity, high mortality, and the associated substantial economic loss [1–3]. *Helicobacter pylori* (*H. pylori*) infection was found to be an important causal factor in the pathogenesis of gastric inflammation and peptic ulcer disease. The World Health Organization defined *H. pylori* as a class I carcinogen for gastric cancer in 1994 [4–6]. *H. pylori* infection gives rise to significant deoxyribonucleic acid damage of epithelial cells and induction of cell cycle dysregulation, all of which are closely associated with significant oncogenic insults in infected mucosa [7–12]. Furthermore, the microorganism surface contains lectins or adhesins, which are capable of recognizing and binding to specific carbohydrate receptors, such as fucose of mucosal epithelial cells [13–15]. They are also capable of producing virulence factors, including vacuolating cytotoxin, which causes epithelium cells degradation and colonizes deeply within the gastric mucus layer [16–18].

**Figure 1.**
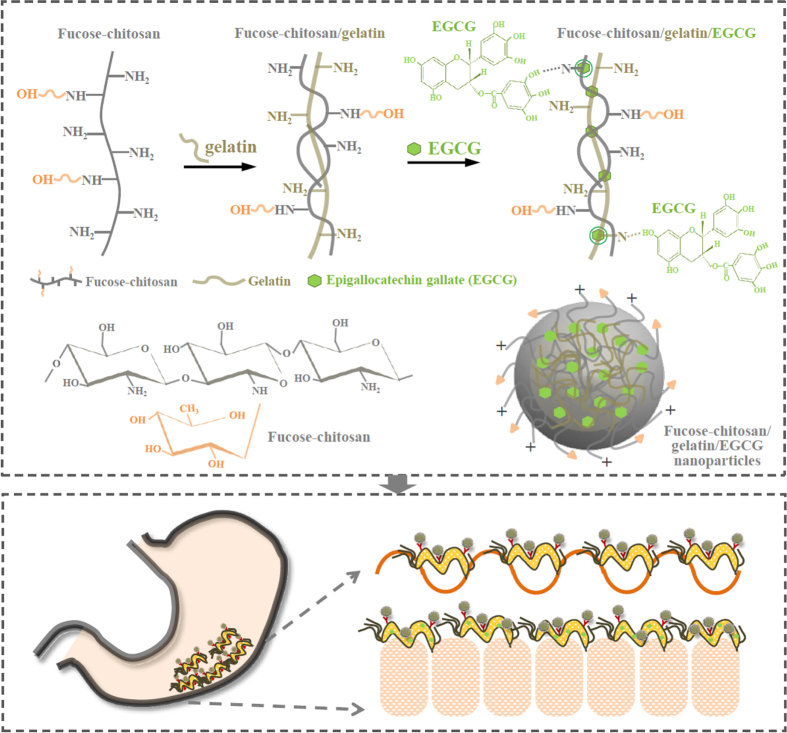
Representation of the prepared fucose–chitosan/gelatin/EGCG nanoparticles and the strategy for eradicating *H. pylori* using nanoparticles.

In our previous study, we developed a fucose-conjugated chitosan nanoparticle encapsulating antibiotic amoxicillin in order to directly contact the region of *H. pylori* on the gastric epithelium and then release amoxicillin to act locally on *H. pylori* at a bactericidal concentration [19]. Amoxicillin is a semisynthetic, orally absorbed, broad spectrum antibiotic which has been extensively used for resistance against *H. pylori* cultures. However, it also has some unpleasant side effects, including a metallic taste in the mouth, diarrhea, and nausea. Such side effects may cause the patient to interrupt the prescribed course of antibiotics [20]. This situation has forced researchers to look for alternative reagents to treat and eradicate *H. pylori* infection. Green tea has a long history of human consumption and could be one of the candidates to attenuate *H. pylori*-induced gastric epithelial injury [21]. The epigallocatechin-3-gallate (EGCG), a major ingredient of green tea, has been revealed to possess antibacterial activity for antiurease activity against *H. pylori* [22, 23]. Therefore, the topical administration of spectrum EGCG through a site-specific is required to attain sufficient bactericidal drug levels in the *H. pylori* infection area for the eradication of *H. pylori* in the gastric mucosa (figure 1).

We prepared nanoparticles composed of fucose–chitosan complex with gelatin of encapsulated EGCG. The fucose–chitosan is a polycationic, non-toxic, mucoadhesive polymer, which allows a prolonged interaction between the delivered drug and the membrane epithelia. Chitosan is a linear polymer of D-glucosamine that is obtained by the alkaline deacetylation of chitin. A number of studies have shown that the charged amino group of D-glucosamine residues in chitosan may interact with *N*-acetylnuraminic acid (sialic acid) in the gastric mucus by electrostatic interaction, which would provide longer residence time in the stomach [24, 25]. Fucose, a deoxyhexose sugar, assists physiologically in modifying various molecules in mammals. The cell surface receptor for *H. pylori* contained fucose residues, and it could be assumed that the affinity of fucose-conjugated nanoparticles toward lectin receptors present on the *H. pylori* surface [26, 27]. This, in turn, facilitates more efficient drug diffusion into the mucus/epithelium layer and corresponding specific receptors of *H. pylori* and may improve *H. pylori* drug therapy [19, 28]. Gelatin is one of the most versatile natural polymers derived from collagen, and it has been widely used in food products and medicines [29]. Gelatin is a biodegradable polymer that contains a large number of glycine residues and proline and 4-hydroxyproline residues, both of which are major amino acids [30–32]. The binding of gelatin by polyphenols is primarily based on hydrogen bonding between hydrophobic amino acid, mostly proline, residues, and phenol rings of polyphenols [33].

In this study, we prepared fucose–chitosan/gelatin/EGCG nanoparticles (figure 1) and examined their physicochemical characteristics using Fourier transform infrared (FTIR) spectroscopy, dynamic light scattering, and transmission electron microscopy (TEM). Their effect of interaction with *H. pylori* were investigated in the human gastric mucosal AGS cell line (human gastric adenocarcinoma cell line) using a confocal laser scanning microscopy (CLSM). We also assessed the *in vivo* experiment to verify the *H. pylori* clearance effect after treating fucose–chitosan/gelatin/EGCG nanoparticles in *H. pylori*-infected mice. Furthermore, histological examination and immunohistochemistry staining were used to detect tissue bacteria density through microscopy in *H. pylori*-infected mouse models.

## 2. Materials and methods

### 2.1. Materials

Chitosan (MW 50 kDa) with approximately 85% deacetylation was obtained from Koyo Chemical (Japan). Fucose (purity ⩾ 99%), EGCG, gelatin, dimethyl sulfoxide, 3-(4,5-dimethyl-thiazol-yl)-2,5-diphenyltetrazolium bromide (MTT), acetic acid, Rhodamine 6G (Rh6G), fluorescein isothiocyanate (FITC), 4′,6-diamidino-2-phenylindole (DAPI), phosphate-buffered saline (PBS), and paraformaldehyde were purchased from Sigma-Aldrich (St Louis, USA). 1,1′-dioctadecyl-3,3,3′,3′-tetramethylindodicarbocyanine-5,5′-disulfonic acid (DilC18(5)-DS) lipophilic dye was from Molecular Probes (Eugene, USA). Fetal bovine serum (FBS), and trypsin–EDTA were from Gibco (Grand Island, New York). All other chemicals and reagents were of analytical grade.

### 2.2. Preparation of fucose–chitosan/gelatin/EGCG nanoparticles

The fucose–chitosan/gelatin/EGCG nanoparticles were prepared by dropping aqueous EGCG into an aqueous fucose–chitosan/gelatin mixed solution. The fucose-conjugated chitosan (fucose–chitosan) was synthesized essentially as described in our previous study [19]. Then, the aqueous fucose–chitosan at concentration (5.0 mg mL^−1^, 0.5 mL) was added by flush mixing with a pipette tip into aqueous gelatin (10.0 mg mL^−1^, 0.5 mL) at fucose–chitosan/gelatin = 2.5 : 5.0 mg mL^−1^. The EGCG solution with distinct concentration (1.25, 2.50, and 5.00 mg mL^−1^, 1.0 mL) was added to 1.0 mL of aqueous fucose–chitosan/gelatin mixed solution and stirred at room temperature for 30 min to form fucose–chitosan/gelatin/EGCG nanoparticles (table 1) at fucose–chitosan/gelatin/EGCG = 1.250 : 2.500 : 0.625, 1.250 : 2.500 : 1.250, 1.250 : 2.500 : 2.500 by mg mL^-1^. The nanoparticles produced were collected by centrifugation for 50 min. The amount of free EGCG in the supernatant was analyzed using high-performance liquid chromatography (HPLC) with a UV detector (Jasco, 875-UV, Tokyo, Japan) and a reversed phase C18 column. It was eluted with acetonitrile—0.005 M citric acid (14 : 86; v/v) at a flow rate of 1.0 mL min^−1^. The EGCG loading efficiency of the nanoparticles was calculated from the following equation:

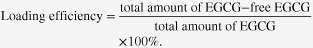



**Table 1.** Effect of different EGCG concentrations on particle sizes, polydispersity indices (PDI), zeta potential values and loading efficiency of the fucose–chitosan/gelatin/EGCG nanoparticles (*n* = 5).

Fucose–chitosan:gelatin:EGCG (mg mL^−1^)	Mean particle size (nm)	Zeta potential (mV)	PDI	Loading efficiency (%)
1.250 : 2.500 : 0.625	213 ± 4	31 ± 3	0.12 ± 0.05	35 ± 2
1.250 : 2.500 : 1.250	230 ± 3	29 ± 3	0.15 ± 0.07	40 ± 1
1.250 : 2.500 : 2.500	244 ± 8	29 ± 3	0.19 ± 0.04	46 ± 2

And, the nanoparticles were resuspended in deionized water, and their size distribution and zeta potential were measured with a zetasizer (Malvern Instruments, Worcestershire, UK). FTIR spectra of the prepared nanoparticles were recorded with a FTIR spectroscopy (Shimadzu Scientific Instruments, USA).

### 2.3. Characterization of the prepared nanoparticles and release profiles of EGCG

The particle size and zeta potential value of the prepared nanoparticles at pH 2.5 (10 mM HCl), pH 6.0 and pH 7.0 (10 mM PBS), simulating environments of the gastric acid, gastric mucosa, or *H. pylori* survival situation, were measured with a zetasizer. The morphology of the prepared nanoparticles was examined by TEM at different pHs. The nanoparticle suspension was placed onto a 400 mesh copper grid coated with carbon. About 2 min after deposition, the grid was tapped with a filter paper to remove surface water and positively stained with an alkaline bismuth solution. Additionally, the continuous EGCG release profiles from EGCG-loaded nanoparticles were investigated in simulated dissolution medium (pH 1.2 for 120 min, then pH 6.0 for another 120 min, and pH 7.0 for the other 300 min) during the whole 540 min in EGCG release experiment at 37 °C. At set time intervals, samples were removed and centrifuged, and the supernatants were subjected to HPLC. The percentage of cumulative amount of released EGCG was determined using a standard calibration curve.

### 2.4. *In vitro H. pylori* growth inhibition and viability of AGS cells studies treated with EGCG solution and fucose–chitosan/gelatin/EGCG nanoparticles

Bacterium *H. pylori* strain 26695 (ATCC 700392) was grown on blood agar plates under microaerophilic conditions for 48–72 h at 37 °C. The colonies were collected and pooled in HBSS solution to an optical density of 1.0 at 590 nm (OD_590_). To characterize the ability of test samples to inhibit *H. pylori* growth, bacterial suspensions were exposed to EGCG solution and fucose–chitosan/gelatin/EGCG nanoparticles with distinct EGCG concentrations (0, 5, 10, 20, and 30 mg L^−1^) for 2 h. After 2 h, the bacteria were centrifuged and incubated in a growth medium for an additional 22 h. The extent of growth inhibition was determined using OD_590_ measurements, and *in vitro* antibacterial activity was quantified by calculating the percentage of growth inhibition, compared to untreated *H. pylori*.

The AGS cell line (ATCC CRL 1739) was cultured with RPMI 1640 medium containing 10% FBS, penicillin (100 U mL^−1^), and streptomycin (100 *μ*g mL^−1^). The AGS cells were seeded at 5 × 10^4^ cells/well in 96-well plates and allowed to adhere overnight. The growth medium was replaced with HBSS solution containing EGCG solution and fucose–chitosan/gelatin/EGCG nanoparticles with EGCG concentrations (0, 5, 10, 20, and 30 mg L^−1^) for 2 h. After 2 h, the test samples were aspirated and the cells washed twice with 100 *μ*L of PBS. The cells were then incubated in growth medium for an additional 22 h. After specific times, the cells were incubated in growth medium containing 1 mg mL^−1^ MTT for an additional 4 h. Dimethyl sulfoxide (100 *μ*L) was added to each well to ensure solubilization of the formed formazan crystals. The optical density was read with a microplate spectrofluorometer (Molecular Devices SpectraMax M2^*e*^, Sunnyvale, USA) at a wavelength of 570 nm. All experiments were performed six times with eight replicate wells for each sample and control per assay [34, 35].

### 2.5. Evaluating the relationship between *H. pylori* and fucose–chitosan/gelatin/EGCG nanoparticles co-culture with AGS cells

To observe the adhesion of *H. pylori* and fucose–chitosan/gelatin/EGCG nanoparticles to cells, the fluorescent bacteria were labeled with DilC18(5)–DS lipophilic dye according to the procedure described in our previous study [19]. The synthesis of FITC-fucose–chitosan was based on the reaction between the free amino groups of fucose–chitosan and the isothiocyanate group of FITC. Briefly, 5 mg of FITC in 5 mL of dehydrated methanol were added to 10 ml of 100 mg fucose–chitosan in deionized water. After 12 h of reaction in the dark at ambient conditions, in order to remove the unconjugated FITC, the FITC-fucose–chitosan solution was then dialyzed for three days in the dark against 5 L of deionized water; the water was replaced on a daily basis. The resultant FITC-fucose–chitosan was lyophilized in a freeze dryer. The Rh6G-EGCG was prepared on the reaction between the Rh6G and the EGCG. EGCG (100 mg) was dissolved completely in 50 mL of deionized water and 2 mg of Rh6G was dissolved completely in 1 mL of acetonitrile. The Rh6G solution was added gradually to the EGCG solution, and then 1 mg of 1-ethyl-3-(3-dimethylaminopropyl) carbodiimide hydrochloride was added with continuous stirring at room temperature for 12 h before being freeze-dried. To remove the unconjugated Rh6G, the dry Rh6G-EGCG sample was dissolved by adding deionized water (50 mL). The precipitate was removed to repeated cycles of washing and centrifugation (6000 rpm for 15 min) until no fluorescence was detected in the precipitate. The fluorescent nanoparticles (FITC-fucose–chitosan/gelatin/Rh6G-EGCG nanoparticles) were prepared as described in the previously mentioned preparation nanoparticles method.

The AGS cells grew on costar transwell plates at a seeding density of 5 × 10^5^ cells/insert and the culture medium was added to both the donor and acceptor compartments. The medium was replaced every 48 h for the first six days and every 24 h thereafter. The cultures were kept in an incubator and were used for 24–30 days after seeding [36]. DiIC18(5)–*H. pylori* was incubated with the AGS cells on transwell for 2 h. The HBSS solution containing the FITC-fucose–chitosan/gelatin/Rh6G-EGCG nanoparticles was then introduced into the donor compartment of the AGS cells for 2 h at 37 °C. After incubation, the test samples were aspirated. The cells were washed twice with PBS and stained with DAPI for 15 min. The stained cells were examined by CLSM with excitation at 340, 488, 543, and 633 nm at 0.5 *μ*m intervals.

### 2.6. *In vivo H. pylori* growth inhibition study and immunohistochemistry staining analysis

All animal care and use complied with the 1996 revision of the ‘Guide for the Care and Use of Laboratory Animals’ prepared by the Institute of Laboratory Animal Resources, National Research Council, and published by the National Academy Press. Healthy and disease-free six-weeks-old male C57BL/6 J mice weighing 25–30 g were individually housed in polycarbonate cages maintained at constant temperature (22 ± 2 °C) and humidity (55%) and used in this study. After overnight fasting, the mice were inoculated with an *H. pylori* suspension of approximately 1 × 10^9^ CFU mL^−1^, using the intragastric gavage method for seven consecutive days. The mice were then randomly divided into groups. Each group contained six mice and received different EGCG formulations with a fixed 30.0 mg kg^−1^ EGCG dose in the form of EGCG solution, fucose–chitosan/gelatin/EGCG nanoparticles, and deionized water for the control group once daily for seven consecutive days. One day after the administration of the final dose, the mice were sacrificed and their stomachs removed and subjected to testing. Each stomach was homogenized with normal sterile saline (3 mL/stomach), from which serial dilutions were plated on blood agar plates under micro-aerophilic conditions for five days at 37 °C. The viable bacterial count for each gastric wall was calculated by counting the number of colonies on the agar plates. We also used urea broth to detect *H. pylori* by identifying the presence of urease. Urease is an enzyme produced by *H. pylori* that hydrolyzes urea to ammonia and carbon dioxide. If the urea in the broth is degraded and ammonia is produced, an alkaline environment is created and the media will turn pink under a spectrophotometer.

For analysis, biopsies were fixed in buffered paraffin and embedded in paraffin wax. The immunohistochemistry staining for *H. pylori* on approximately 5 *μ*m tissue slides were deparaffinized with xylene and alcohol and then rehydrated in water. Antigen retrieval was performed with a 10 mM sodium citrate buffer at pH 6.0 in a pressure cooker for 10 min. After washing in PBS, the tissue section was also subjected to immunohistochemical analyses using the rabbit polyclonal *H. pylori* antibody (Zytomed Systems GmbH, Germany) and the NovoLink polymer detection system (Leica Biosystems Newcastle, United Kingdom) with 3,3-diaminobenzidine-4HCl as the chromogen. The stained sections of each test sample were then examined at 400× and 1000× magnifications under a light microscope. For histology analysis, a gastric tissue biopsy was performed using light microscopy.

### 2.7. Statistical analysis

Statistical analysis of the differences in the measured properties of the groups was performed with one-way analysis of variance and the determination of confidence intervals, with the statistical package Statistical Analysis System, version 6.08 (SAS Institute, Cary, NC). All data are presented as means and standard deviations, indicated as ‘mean ± SD’. Differences were considered to be statistically significant when the *P* values were less than 0.05.

## 3. Results and discussion

### 3.1. Preparation and characterization of fucose–chitosan/gelatin/EGCG nanoparticles

The fucose–chitosan/gelatin/EGCG nanoparticles were produced by the gelation of aqueous EGCG solution mixed with fucose–chitosan/gelatin mixture solution. As shown in table 1, our fucose–chitosan/gelatin/EGCG nanoparticles were prepared with different EGCG concentrations with the range particle sizes of 210–250 nm and positive zeta potentials, depending on the fucose–chitosan used. We used fucose–chitosan/gelatin to encapsulate EGCG at various concentrations. The EGCG percentage loading efficiency was as follows: 35 ± 2% (for EGCG 0.625 mg mL^−1^), 40 ± 1% (for EGCG 1.250 mg mL^−1^), and 46 ± 2% (for EGCG 2.500 mg mL^−1^), respectively. Therefore, for an EGCG concentration of 2.500 mg mL^−1^, the particle size is 244 ± 8 nm with an appreciably narrower distribution (polydispersity index (PDI) 0.19 ± 0.04), a zeta potential of 29 ± 3 mV, and loading efficiency of 46 ± 2%. PDI is a parameter to define particle size distribution of nanoparticles and it is a dimensionless number extrapolated from the autocorrelation function and ranges from 0 to 1. Values close to 0 indicate a homogeneous dispersion while those greater than 0.3 indicate high heterogeneity [37]. Therefore, EGCG 2.500 mg mL^−1^ with higher loading efficiency and better particle size/distribution was a relatively desired concentration for the subsequent experiments.

**Figure 2.**
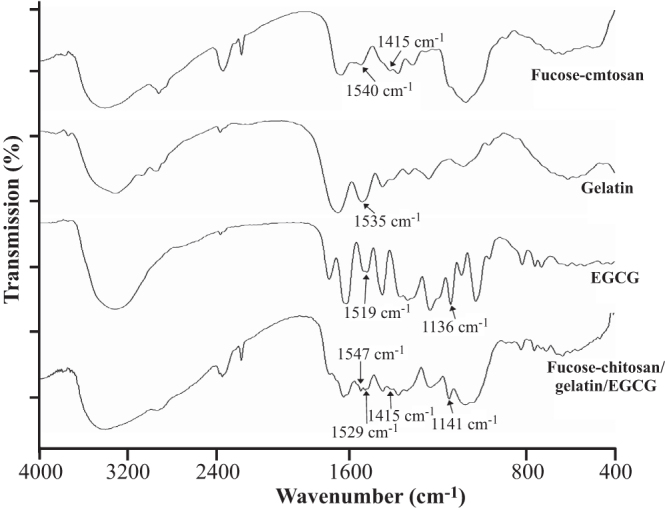
FTIR spectra are vertically shifted for clarity of fucose–chitosan, gelatin, EGCG, and fucose–chitosan/gelatin/EGCG nanoparticles.

Figure 2 shows the FTIR spectra of the fucose–chitosan, gelatin, EGCG, and the fucose–chitosan/gelatin/EGCG nanoparticles. For fucose–chitosan, the spectra showed transmission peaks at 1540 cm^−1^ to protonated amino groups (−NH_3_^+^) on chitosan and at 1415 cm^−1^ to the –CH_3_ bending vibration of fucose. Furthermore, as shown in the spectrum of gelatin and EGCG, the characteristic peaks observed at 1535 cm^−1^ was amide II (N–H bending vibration) on gelatin and 1136 and 1519 cm^−1^ were assigned to C–OH alcohols and C = C aromatic ring on EGCG. In the spectrum of fucose–chitosan/gelatin/EGCG complex, the characteristic peak at 1540 cm^−1^ for −NH_3_^+^ on chitosan and 1535 cm^−1^ for amide II on gelatin disappeared. In its place, a new peak at 1547 cm^−1^ and 1529 cm^−1^ emerged and the characteristic peak of C–OH deformation on EGCG at 1136 cm^−1^ shifted to 1141 cm^−1^. These observations can be attributed to the hydrogen-bond interactions between N atoms in gelatin or chitosan chains and H atoms in hydroxyl groups of EGCG (C–N⋯HO–C), which thus resulted in fucose–chitosan, gelatin, and EGCG complexes that segregated into colloidal nanoparticles (figure 1).

### 3.2. Characterization of the prepared nanoparticles and release profiles of EGCG

**Figure 3.**
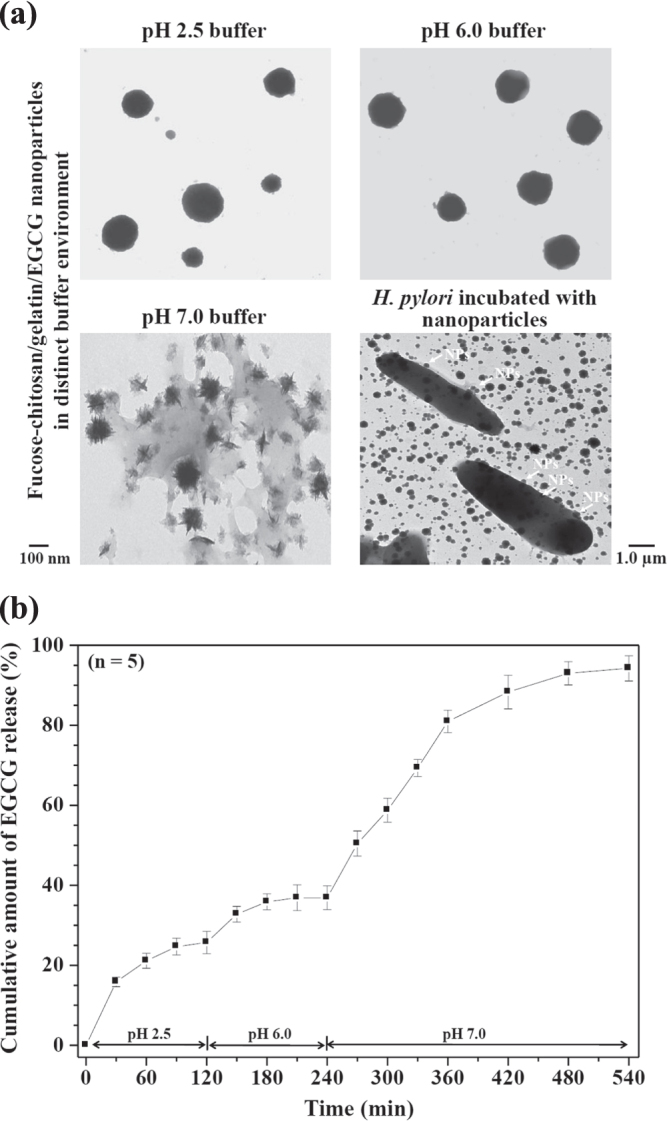
(a) TEM micrographs of the fucose–chitosan/gelatin/EGCG nanoparticles in specific pH environments and *H. pylori* morphology after treatment with fucose–chitosan/gelatin/EGCG nanoparticles. (b) *In vitro* release profiles of EGCG from fucose–chitosan/gelatin/EGCG nanoparticles at different pH values at 37 °C (*n* = 5).

Figure 3 shows the nanoparticle morphology and release profiles of EGCG of fucose–chitosan/gelatin/EGCG nanoparticles at distinct pH values. As shown in figure 3(a), the prepared nanoparticles were independent stay at each pH value (pH 2.5, pH 6.0, and pH 7.4) for 120 min and observed their morphology using TEM. At pH 2.5 (simulating the pH of gastric acid), the morphology particle size became slightly swollen, leading to a little burst of EGCG release effect. The proportion of EGCG released from the nanoparticles was a significant release of 26 ± 3% for 120 min (figure 3). At pH 6.0 (simulating gastric mucosa situation), only a very small amount of EGCG was released from the prepared nanoparticles. We prepared the nanoparticles composed of fucose–chitosan complex with gelatin of encapsulated EGCG. The fucose–chitosan is a mucoadhesive polymer, which allows a prolonged interaction between the delivered drug and the membrane epithelia, and then targets the region of *H. pylori* on the gastric epithelium [19]. Furthermore, at pH 7.0 (simulating *H. pylori* survival situation), the chitosan has a pKa value around 6.5 [3], and thus the chitosan was deprotonated, leading to the collapse of a part of the nanoparticles (figure 3(a)). Meanwhile, the fucose–chitosan/gelatin/EGCG complex can be attributed to the hydrogen-bond interactions between N atoms in gelatin or chitosan chains and H atoms in hydroxyl groups of EGCG (figure 1), due to the EGCG’s flow cumulative release from 59 ± 3% (in 60 min at pH 7.0) to 94 ± 3% (in 300 min at pH 7.0) (figure 3(b)).

### 3.3. *In vitro H. pylori* growth inhibition and viability of AGS cells treated with EGCG solution and fucose–chitosan/gelatin/EGCG nanoparticles

**Figure 4.**
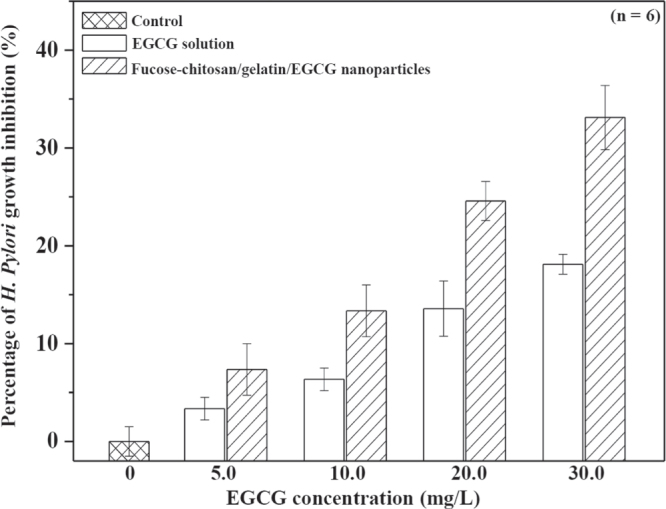
Percentage of *H. pylori* growth inhibition for EGCG solution and fucose–chitosan/gelatin/EGCG nanoparticles (*n* = 6).

**Figure 5.**
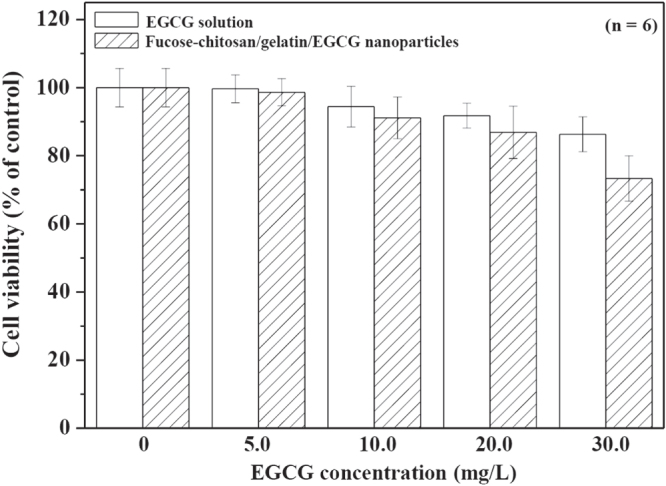
AGS cell viability for EGCG solution and fucose–chitosan/gelatin/EGCG nanoparticles (*n* = 6).

The *H. pylori* is known to be an etiological agent of chronic gastritis and peptic ulcer as well as a risk factor for the development of gastric cancer [38, 39]. Current triple therapy with antibiotics and a proton pump inhibitor shows a high eradication rate and a low incidence of harmful side effects, including non-compliance and occurrence of resistance to antibiotics [40]. Green tea could be one of the candidates to attenuate *H. pylori*-induced gastric epithelial injury [21]. EGCG, a major ingredient of green tea, has been revealed to have antibacterial activity for antiurease activity against *H. pylori* [22, 23] and significantly reduced interleukin-1*β* (IL-1*β*), IL-8, and interferon-*γ*-induced nitric oxide and cyclo-oxygenase-2 production through the inhibition of the NF-*κ*B signal pathway, [41–43] all of which are also pathogenically implicated in the perpetuated gastric inflammation caused by *H. pylori*. Figure 4 shows the percentage of *H. pylori* growth inhibition resulting from treatment with various EGCG concentrations (0, 5, 10, 20, and 30 by mg L^-1^) for 24 h. In 20 and 30 mg L^−1^ of EGCG, significant growth inhibition, 14 ± 3% and 18 ± 1%, was observed compared to the control (*p* < 0.05). *H. pylori* has a hydrophilic property and its outer membrane is composed of a lipopolysaccharide structure with a negatively charged surface [44, 45]. The fucose–chitosan/gelatin/EGCG nanoparticles featured a positive surface charge and mucoadhesive properties, which allowed a prolonged interaction between the delivered EGCG and the *H. pylori*. Furthermore, this also increased the values of *H. pylori* growth inhibition (25 ± 2% to 33 ± 3% at EGCG 20–30 mg L^−1^ concentrations). Therefore, the EGCG-loaded nanoparticles significantly increase the values of *H. pylori* growth inhibition compared to EGCG solution (figure 4). The cytotoxicity of various concentrations of EGCG solution using AGS cells was also investigated (figure 5). Cell viability was generally not affected by the pure EGCG and fucose–chitosan/gelatin/EGCG nanoparticle solutions at EGCG concentrations below 20.0 mg L^−1^; however, it declined slightly when exposed to increasing concentrations at 30 mg L^−1^. Previous studies demonstrated that EGCG, the most abundant and active component of green tea, has chemopreventive and chemotherapeutic properties for a variety of cancers. Such properties inhibited tumor growth by anti-angiogenesis and by inhibiting proliferation and inducing apoptosis [46]. To track the cellular internalization of the nanoparticles and *H. pylori* without damaging the cultured cells, we used EGCG at 20.0 mg L^−1^ concentration to treat *H. pylori* infection and track cellular internalization of particles without causing damage to the cultured cells.

### 3.4. Relationship between *H. pylori* and fucose–chitosan/gelatin/EGCG nanoparticles co-culture with AGS cells

**Figure 6.**
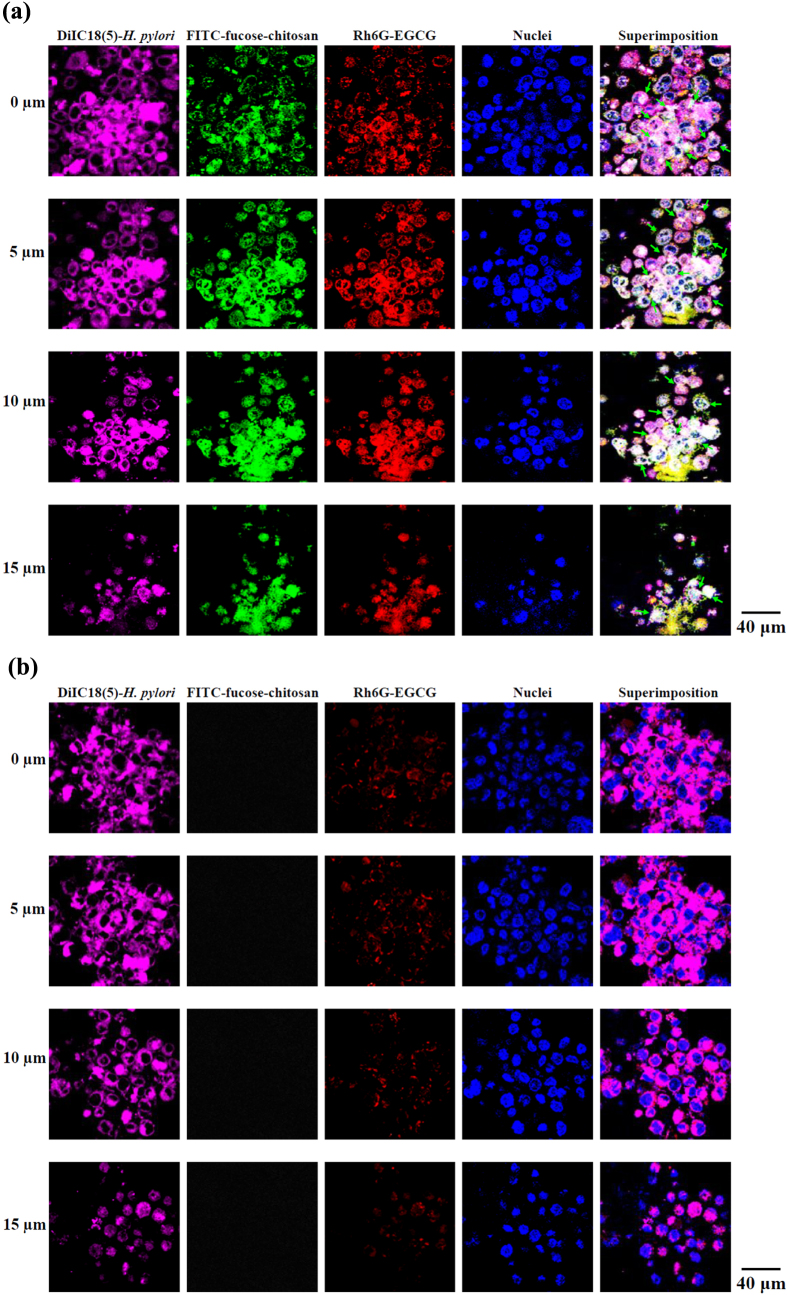
Fluorescence images of AGS cell monolayers infected with *H. pylori* and incubated for 2 h with (a) fucose–chitosan/gelatin/EGCG nanoparticles and (b) only EGCG solution.

Oral administration is considered the most efficient delivery system; however, the oral EGCG is not good because of its poor stability and the fact that it cannot reach the targeted sites of the antibacterial activity [47]. To study the interaction of EGCG solution and EGCG-loaded nanoparticles with *H. pylori* attached to the epithelia, we developed a system of fluorescent DiIC18 (5)–*H. pylori* infecting AGS monolayers, which were then treated with fluorescent FITC-fucose–chitosan/gelatin/Rh6G-EGCG nanoparticles. As shown in figure 6, after 2 h of infection, the AGS-adapted DilC18(5)–*H. pylori* (purple spot) preferentially cell–cell junctions and was observed within the cells. The AGS cell monolayers were incubated with fluorescent nanoparticles (FITC-fucose–chitosan: green spot, Rh6G-EGCG: red spot) and observed by CLSM whether the nanoparticles and EGCG were co-localized and interacted at the same location of intercellular spaces whether the cell cytoplasm of *H. pylori* infected sites at different depths (green arrows; figure 6(a)). By contrast, cells incubated with Rh6G-EGCG solution alone (red spot) showed less obvious fluorescence signals in intercellular spaces (figure 6(b)) than those seen after incubation with EGCG loaded in nanoparticles. The fucose–chitosan/geleatin/EGCG nanoparticles produced an intense fluorescence that emanated from deep within the cells, indicating that the nanoparticles were capable of carrying EGCG to AGS cell infected with *H. pylori.*

### 3.5. *In vivo H. pylori* growth inhibition study

**Figure 7.**
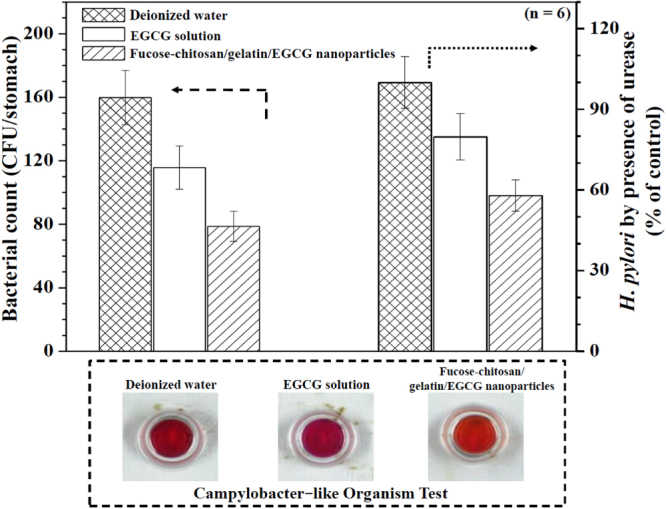
Effects of deionized water, EGCG solution alone, and fucose–chitosan/gelatin/EGCG nanoparticles in a mouse model with *H. pylori*-induced gastric infection (*n* = 6).

*H. pylori* produce the enzyme urease, which hydrolyzes urea to ammonia and carbon dioxide; these products in turn neutralize stomach acid and raise pH levels to allow successful colonization of the gastric environment by *H. pylori* [48]. Some pharmacological studies have demonstrated that EGCG inhibits the urease activity and motility of *H. pylori*, which may contribute to its antibacterial activity *in vivo* [22]. Figure 7 shows the *in vivo* clearance data of *H. pylori* infection after administration of 30 mg kg^−1^ EGCG solution for seven consecutive days. The mean bacterial count of the control group of mice that was given only sterile water was 160 ± 17 (CFU/stomach). Treatment with EGCG solution alone resulted in a mean bacterial count of 116 ± 14 (CFU/stomach). Our prepared positively fucose–chitosan/gelatin/EGCG nanoparticles were also pH-sensitive and mucoadhesive, which could help avoid both the EGCG from being quickly released from the nanoparticles (figure 3) and prolonged interaction between the delivered drug and the membrane epithelia. In the particular case of eradication, treatment with 30 mg kg^−1^ EGCG in fucose–chitosan/gelatin/EGCG nanoparticles gave a mean bacterial count of 79 ± 10 (CFU/stomach) and significantly increased the inhibitory effects on *H. pylori* infected-mice compared with EGCG solution alone. After conducting the unease test, the values for *H. pylori* were found to decrease only slightly, at a count of 80 ± 9% (only EGCG solution) and significantly 58 ± 6% (fucose–chitosan/gelatin/EGCG nanoparticles), compared to the control values at 100%. In the presence of *H. pylori* urease, urea is converted into ammonium hydroxide and the color of the indicator changed from yellow to red. Figure 7 also shows that the mice infected with *H. pylori* and administered sterile water indicated a positive response as the media changed colors to red after a campylobacter-like organism test. Following treatment with 30 mg kg^−1^ EGCG, the form of EGCG solution alone and fucose–chitosan/gelatin/EGCG nanoparticles, the media changed color to a reddish-orange and light orange, respectively. Therefore, to increase the clinical treatments, the positively fucose–chitosan/gelatin/EGCG nanoparticles was investigated.

### 3.6. Immunohistochemistry staining *H. pylori* analysis

**Figure 8.**
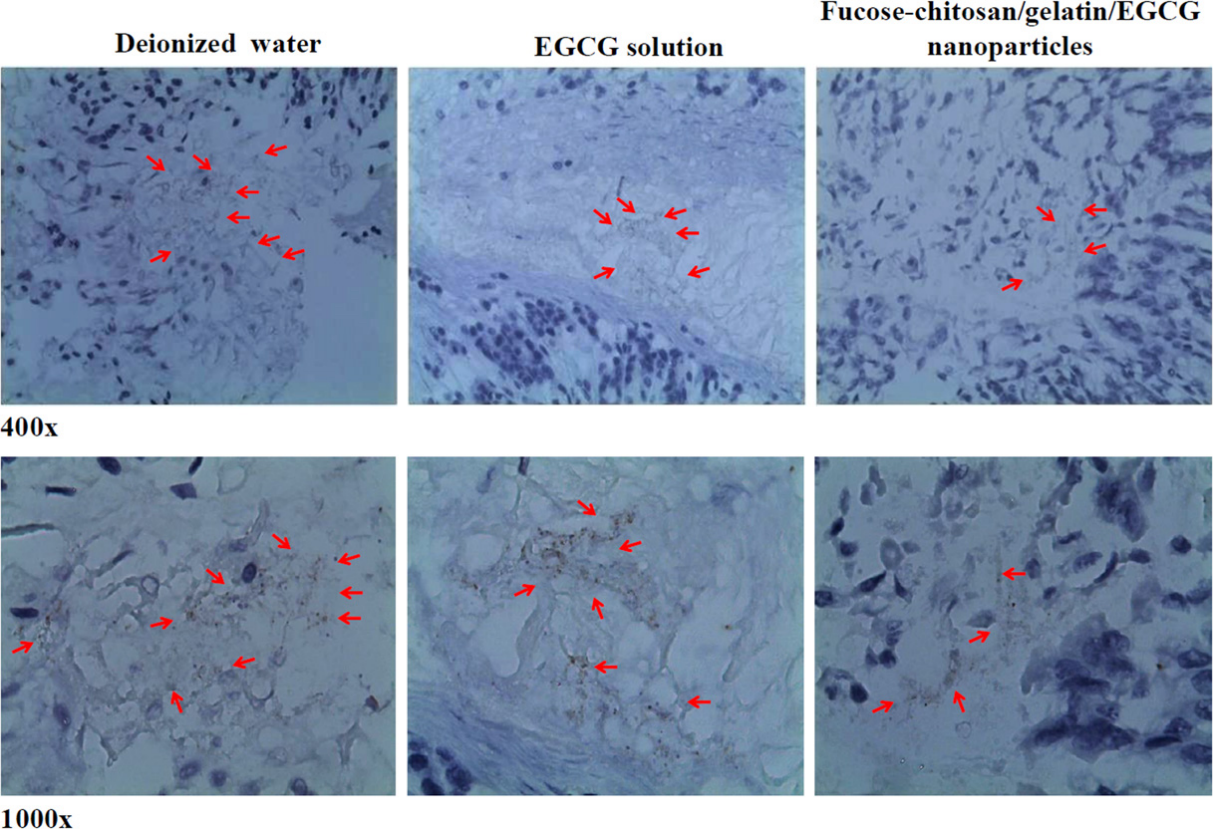
Immunohistochemical staining analysis of a *H. pylori*-infected mouse treated with deionized water, EGCG solution alone, and fucose–chitosan/gelatin/EGCG nanoparticles.

Figure 8 shows the spiral *H. pylori* in the gastric mucosa, as visualized through immunohistochemistry staining in a representative mouse stomach after inoculation. The immunostained slide of the gastric tissue biopsy of infected mice treated with deionized water showed a high level of bacterial colonization on top of the mucous layer; furthermore, epithelial cells with some bacteria appeared to be in luminal spaces between villus projections (brown color; red arrows). Treatment with EGCG solution showed the presence of bacteria in close contact with epithelial cells and that numerous bacteria in the glandular lumina had penetrated deeply into the gastric glands (red arrows) at 400× and 1000× magnifications. Umamaheswari and Jain applied fucose-specific lectin-conjugated gliadin particles that could plug and seal the carbohydrate receptors at the site of *H. pylori* infection [49]. Our prepared nanoparticles composed fucose–chitosan polymer which corresponded to specific receptors of *H. pylori* and facilitated more efficient drug diffusion into the mucus/epithelium layer to improve *H. pylori* drug therapy. The density of bacteria in the gastric tissue biopsy in infected mice treated with our prepared fucose–chitosan/gelatin/EGCG nanoparticles was obviously less than that observed in the slides for the other groups (red arrows). Therefore, the results clearly indicate that the fucose–chitosan/gelatin/EGCG nanoparticles are effective in reducing *H. pylori*-associated gastric bacterial colonization in *H. pylori-*infected animal models.

## 4. Conclusions

The fucose–chitosan/gelatin developed an efficient carrier for EGCG delivery and directly made contact with the *H. pylori* region on the gastric epithelium. *In vitro* drug release analysis of the nanoparticles indicated that the system could control EGCG release in a simulated gastrointestinal dissolution medium and interacted specifically with the intercellular space at the site of *H. pylori* infection. *In vivo* clearance assays indicated that the fucose–chitosan/gelatin/EGCG nanoparticles had significantly inhibited *H. pylori* growth and effectively reduced *H. pylori*-associated gastric inflammation in the gastric-infected mouse model.

## References

[C1] Türkdoğan M K, Hekim H, Tuncer İ, Aksoy H (1999). East. J. Med..

[C2] van Doorn L J, Figueiredo C, Sanna R, Plaisier A, Schneeberger P, de Boer W, Quint W (1998). Gastroenterology.

[C3] Lin Y H, Chang C H, Wu Y S, Hsu Y M, Chiou S F, Chen Y J (2009). Biomaterials.

[C4] Uemura N, Okamoto S, Yamamoto S, Matsumura N, Yamaguchi S, Yamakido M, Taniyama K, Sasaki N, Schlemper R J (2001). New. Eng. J. Med..

[C5] Hahm K B, Lee K J, Kim J H, Cho S W, Chung M H (1998). Dig. Dis. Sci..

[C6] Rao Y K, Lien H M, Lin Y H, Hsu Y M, Yeh C T, Chen C C, Lai C H, Tzeng Y M (2012). Food Chem..

[C7] O’rourke E J, Chevalier C, Pinto A V, Thiberge J M, Ielpi L, Labigne A, Radicella J P (2003). Proc. Natl. Acad. Sci. USA.

[C8] Bagchi D, McGinn T R, Ye X, Bagchi M, Krohn R L, Chatterjee A, Stohs S J (2002). Dig. Dis. Sci..

[C9] Moss S F, Calam J, Agarwal B, Wang S, Holt P R (1996). Gut.

[C10] Ahmed A, Smoot D, Littleton G, Tackey R, Walters C S, Kashanchi F, Allen C R, Ashktorab H (2000). Microbes Infect..

[C11] Mannick E E, Bravo L E, Zarama G, Realpe J L, Zhang X J, Ruiz B, Fontham E T, Mera R, Miller M J, Correa P (1996). Cancer Res..

[C12] Fan X, Crowe S E, Behar S, Gunasena H, Ye G, Haeberle H, Van Houten N, Gourley W K, Ernst P B, Reyes V E (1998). J. Exp. Med..

[C13] Borlace G N, Butler R N, Brooks D A (2008). Helicobacter.

[C14] Fedwick J P, Lapointe T K, Meddings J B, Sherman P M, Buret A G (2005). Infect. Immun..

[C15] Falk P, Roth K A, Borén T, Westblom T U, Gordon J I, Normark S (1993). Proc. Natl. Acad. Sci. USA.

[C16] Lin Y H, Chiou S F, Lai C H, Tsai S C, Chou C W, Peng S F, He Z S (2012). Process Biochem..

[C17] van Amsterdam K, van Vliet A H, Kusters J G, van der Ende A (2006). FEMS Microbiol. Rev..

[C18] Cover T L, Blaser M J (1992). J. Biol. Chem..

[C19] Lin Y H, Tsai S C, Lai C H, Lee C H, He Z S, Tseng G C (2013). Biomaterials.

[C20] Chang C H, Huang W Y, Lai C H, Hsu Y M, Yao Y H, Chen T Y, Wu J Y, Peng S F, Lin Y H (2011). Acta Biomater..

[C21] Graham H N (1992). Prev. Med..

[C22] Mabe K, Yamada M, Oguni I, Takahashi T (1999). Antimicrob. Agents Chemother..

[C23] Yee Y K, Koo M W (2000). Aliment. Pharmacol. Ther..

[C24] Lehr C M, Bouwstra J A, Schacht E H (1992). Int. J. Pharm..

[C25] Miyazaki S, Nakayama A, Oda M, Takada M, Attwood D (1994). Biol. Pharm. Bull..

[C26] Liu T W, Ho C W, Huang H H, Chang S M, Popat S D, Wang Y T, Wu M S, Chen Y J, Lin C H (2009). Proc. Natl. Acad. Sci. USA.

[C27] Rajinikanth P S, Mishra B (2007). Acta Pharm..

[C28] Mi F L, Sung H W, Shyu S S (2000). J. Polym. Sci. A.

[C29] Lin Y H, Lin J H, Peng S F, Yeh C L, Chen W C, Chang T L, Liu M J, Lai C H (2011). J. Appl. Polym. Sci..

[C30] Pal K, Banthia A K, Majumdar D K (2007). J. Mater. Sci., Mater. Med..

[C31] Petrenko Y A, Ivanov R V, Petrenko A Y, Lozinsky V I (2011). J. Mater. Sci., Mater. Med..

[C32] Chen K Y, Yao C H (2011). BioMedicine.

[C33] Shutava T G, Balkundi S S, Vangala P, Steffan J J, Bigelow R L, Cardelli J A, O’neal D P, Lvov Y M (2009). ACS Nano.

[C34] Huang W Y, Yeh C L, Lin J H, Yang J S, Ko T H, Lin Y H (2012). J. Mater. Sci., Mater. Med..

[C35] Lin Y H, Lin J H, Wang S H, Ko T H, Tseng G C (2012). J. Biomed. Mater. Res. B.

[C36] Kim J S, Kim J M, Jung H C, Song I S (2001). Dig. Dis. Sci..

[C37] Ahlin P, Kristl J, Kristl A, Vrecer F (2002). Int. J. Pharm..

[C38] Yanagawa Y, Yamamoto Y, Hara Y, Shimamura T (2003). Curr. Microbiol..

[C39] Blaser M J (1987). Gastroenterology.

[C40] Misiewicz J J, Harris A W, Bardhan K D, Levi S, O’morain C, Cooper B T, Kerr G D, Dixon M F, Langworthy H, Piper D (1997). Gut..

[C41] Katiyar S K, Challa A, McCormick T S, Cooper K D, Mukhtar H (1999). Carcinogenesis.

[C42] Trompezinski S, Denis A, Schmitt D, Viac J (2003). Arch. Dermatol. Res..

[C43] Ahmed S, Rahman A, Hasnain A, Lalonde M, Goldberg V M, Haqqi T M (2002). Free Radical Biol. Med..

[C44] Smith J I, Drumm B, Neumann A W, Policova Z, Sherman P M (1990). Infect. Immun..

[C45] Moran A P, Helander I M, Kosunen T U (1992). J. Bacteriol..

[C46] Zhu B H, Chen H Y, Zhan W H, Wang C Y, Cai S R, Wang Z, Zhang C H, He Y L (2011). World J. Gastroenterol..

[C47] Hu B, Ting Y, Zeng X, Huang Q (2013). J. Agric. Food Chem..

[C48] Scott D R, Weeks D, Hong C, Postius S, Melchers K, Sachs G (1998). Gastroenterology.

[C49] Umamaheshwari R B, Jain N K (2003). J. Drug Targeting.

